# CABG versus PCI: What is the optimal strategy for multi-vessel disease?

**DOI:** 10.1016/j.amsu.2022.104354

**Published:** 2022-08-18

**Authors:** Ayush Kumar, Priya, Govinda Khatri, Mohammad Mehedi Hasan

**Affiliations:** a Aga Khan University Hospital, Karachi, Pakistan; b Dow University of Health Sciences, Karachi, Pakistan; c Department of Biochemistry and Molecular Biology, Faculty of Life Science, Mawlana Bhashani Science and Technology University, Tangail, Bangladesh

Multi-vessel coronary artery disease (CAD) is a pathological disease in which at least two or three of the epicardial coronary arteries have significant atherosclerosis. The multivessel disease is typically associated with an increased prevalence of comorbidities, left ventricular failure, and cardiovascular events [1]. Re-vascularization is a common procedure used to improve the clinical outcome of patients. Percutaneous coronary intervention (PCI) and coronary artery bypass grafting are two revascularization techniques for people with multivessel CAD (CABG). However, the best re-vascularization strategy is still uncertain. Several randomized controlled trials (RCTs) and retrospective studies were conducted to assess the results and risk/benefit ratios of the two most commonly used CAD treatments, percutaneous coronary intervention (PCI) and coronary artery bypass grafting (CABG) surgery. The SYNTAX research included 1800 patients who were assessed for PCI non-inferiority versus CABG. The non-inferiority criterion was not met because the PCI group had significantly higher rates of serious adverse cardiac or cerebrovascular events at 12 months (17.8%, vs. 12.4% for CABG) [2,3]. The CARDia (Coronary Artery Revascularization in Diabetes) study was the first to look into CAD therapy in a small group of diabetic patients, and it found that CABG was advantageous, with cumulative mortality risk, MI, stroke, and recurrent revascularization rates of 11.3% in the CABG group and 19.3% in the PCI group at 1 year [4]. The FREEDOM study, which included 1900 patients with complicated MVD and diabetes, confirmed these findings, revealing that the PCI group had considerably lower 5-year rates of a composite outcome, which comprised mortality from any cause, nonfatal MI, or nonfatal stroke (26.6% vs. 18.7% in the CABG group). Despite the fact that the CABG cohort had a greater risk of stroke, the PCI group had substantially higher mortality and MI, suggesting that CABG might benefit the diabetic population more than PCI [5]. Sipahi et al. conducted a meta-analysis of 6 RCTs including 6055 patients and discovered that, when compared to PCI, CABG significantly reduced overall mortality, myocardial infarction, and recurrent revascularization. Although there was an increase in strokes following CABG, it was not statistically significant [6]. Fanari and colleagues recently released insightful research in which they performed a meta-analysis of six RCTs and assessed the long-term follow-up outcomes. In comparison to CABG, PCI had a significantly greater rate of target vessel revascularization, a lower incidence of stroke, and no difference in mortality or MI after one year. However, after 5 years, PCI was linked to an increased risk of mortality and MI. Diabetics had the highest death rate in the PCI group [7]. Benedetto et al. performed a meta-analysis on five randomized studies with a total of 4563 participants. After an average of 3.4 years of follow-up, drug-eluting stent-PCI was associated with a significantly increased risk of overall mortality, MI, and repeat revascularization. CABG increased the risk of stroke somewhat. CABG significantly reduced the risk of all-cause death (3.3%) and myocardial infarction (4.3%). The use of DES-PCI reduced the absolute risk of stroke (0.9%) [8]. In a recent meta-analysis of 11 randomized studies including 11 518 patients, Head et al. discovered that CABG outperformed PCI in patients with multivessel disease, particularly those with diabetes and greater coronary complexity. In people with left main illness, however, there was no difference between CABG and PCI [9]. According to the data, CABG remains the best revascularization procedure for MVD, with decreased mortality and risk of recurrent revascularization. In terms of long-term survival, the total risk of stroke associated with CABG does not exceed the advantages of this revascularization technique. The results gained from this study should be carefully addressed when translating the findings to real-world circumstances, which commonly contain various case mixes with numerous comorbidities.

## Ethical approval

NA.

## Please state any sources of funding for your research

NA.

## Author contribution

All authors meet the inclusion criteria, and all authors read and approved the final version of the manuscript.

## Registration of research studies

1. Name of the registry: NA
2. Unique Identifying number or registration ID: NA
3. Hyperlink to your specific registration (must be publicly accessible and will be checked): NA

## Guarantor

Mohammad Mehedi Hasan, Department of Biochemistry and Molecular Biology, Faculty of Life Science, Mawlana Bhashani Science and Technology University, Tangail, 1902, Bangladesh. Email: mehedi.bmb.mbstu@gmail.com.

## Consent

NA.

## Declaration of competing interest

NA.
